# Preventive dental care utilization for children with special healthcare needs including the COVID-19 pandemic, national survey of children’s health, 2020

**DOI:** 10.1371/journal.pgph.0001540

**Published:** 2023-02-21

**Authors:** R. Constance Wiener

**Affiliations:** Department of Dental Public Health and Professional Practice, West Virginia University School of Dentistry, Morgantown, West Virginia, United States of America; Indian Institute of Technology Bombay, INDIA

## Abstract

It is important to determine access to preventive care among vulnerable populations. The purpose of this research is to compare preventive dental utilization between children with special healthcare needs (CSHCN) and children without special healthcare needs (CWSHCN) using National Survey of Children’s Health, 2020 (NSCH, 2020) data. A cross-sectional study design was used in this secondary data analysis of data from June 2020 to January 2021, NSCH, 2020, a publicly available data set with demographic and healthcare information. Parents/guardians responded to validated questions including one asking them to recall preventive dental services received during the previous year (June 2019 to January 2021). This was a critical time of transition from normal, pre-pandemic activities (June 2019 to March 2020) to the pandemic period (March 2020 to January 2021) with restrictions and no available vaccinations for children. Data analyses for the NSCH, 2020 data included frequency determinations, Chi Square analyses by preventive dental visit, and logistic regression analyses. There were 40,140 children in the sample, and 20.6% were CSHCN. Overall, 75.0% of children received a dental preventive visit. This study had an interaction of CSHCN status and medical visits within the previous year in which having had a medical visit was associated with CSHCN status also having a dental visit; while the CSHCN who did not have a medical visit were less likely to have a dental visit in unadjusted analysis. However, the pattern for CSHCN and the medical visit reversed in adjusted analysis. In adjusted analysis, CWSHCN and a medical visit were the most likely to also have a dental visit during this period. Many factors influenced access to preventive dental care in the months leading up to and including the COVID-19 pandemic. CSHCN with or without a medical visit were less likely to have preventive dental care than CWSHCN with a medical visit in logistic regression analysis adjusted for sex, race/ethnicity, age, smoking in the household, medical visits within the year, insurance coverage, and highest level of education in the household.

## Introduction

In 2010, 78.9% of children, ages ≥2 to <18 years, had a dental visit in the past year [1]. This increased to 85.9% in 2018 [2]. Since 2018, data acquisition by nationally-recognized, reputable sources has been difficult due to the COVID-19 pandemic. However, the National Survey of Children’s Health, 2020 (NSCH, 2020) researchers were able to conduct their research during the pandemic by changing data collection and sampling frame procedures. Additionally, 90.4% of respondents completed the topical NSCH, 2020 survey *online* compared to 75.9% in 2017 [2]. The researchers provided potential explanation for the overwhelming online use as being due to online experience with responding to the 2020 Census, and the need to conduct many life activities online due to theCOVID-19 pandemic [2]. Although they noted marginally higher responses from Hispanic and non-Hispanic black participants in 2020, after data cleaning they did not find substantial changes in response distributions [2]. For the NSCH, 2020 survey, the researchers asked questions corresponding to healthcare received from June 2019 to January 2021. This was the time just before the COVID-19 pandemic through the period with COVID-19 restrictions and no available vaccinations for youth. Many healthcare-seeking behaviors transitioned during this time. Researchers indicated that the impact of the COVID-19 lockdowns and isolation created increases in anxiety, depression, anger, and other healthcare needs among children [3]. COVID-19 was also associated with greater healthcare needs particularly for children with developmental disabilities [4] and children with other special healthcare needs [5].

Although needs increased, many in-person services were limited or not available. At any given time, children with special healthcare needs (CSHCN) are among the people at increased risk or require more care for physical, developmental, behavioral, or emotional needs than their typically developing peers—children without healthcare needs (CWSHCN) [6].

The CDC estimates that 20% of U.S. children are CSCHN [6]. CSHCN status is not based entirely on lists of conditions/diseases, or functional status [7]; however, several long-standing medical conditions are recognized as having special healthcare needs (ie., asthma and diabetes) [6]. Over the years, researchers reported many healthcare challenges for CSHCN. As some examples, children with autism spectrum disorders had nearly 4 times higher odds of unmet healthcare needs compared to CWSHCN [8]; children with cerebral palsy had 2.5 as high odds of mental health disorders as children without cerebral palsy [9]; and children with Attention-Deficit/Hyperactivity Disorder and Tourette Syndrome had 11.6% unmet healthcare need [10]. Nevertheless, researchers, using data from 2016–2018, reported 84% of U.S. CSHCN had preventive dental services as compared with 78% of CWSHCN (*p* < .0001) [11].

Dental utilization for CSHCN and CWSHCN from June 2019 to January 2021, the transition period before the pandemic (June 2019 to March 2020) through the period with restrictions and no available vaccinations for youth (March 2020 to January 2021), is largely unknown. There is a potential differential on access to care between CSHCN and CWSHCN, although many patients, regardless of special needs status, avoided dental visits due to fear of contracting COVID-19 [12], numerous and controversial recommendations [13], and initial treatment limitations. Researchers indicated that there was less access primarily among socially disadvantaged groups [14] primarily racial and ethnic minorities [12] during this time.

It is important to understand the patterns of dental care utilization, particularly for vulnerable populations in times of disruption. Overall, the U.S. population of Medicaid beneficiaries had an initial drop in dental visits of 14.5% and an overall net decrease of 12.1% in dental services during the initial COVID-19 surge [15]. Preventive and diagnostic services had the largest decreases that were not caught up [15]. Researchers reported concerns that the missed visits would result in more short and long-term consequences including an increased use of emergency departments (ED). The projected increase in ED use due to dental issues is expected to be a 0.14–0.20% increase [15]. Understanding the patterns will help in resource allocation and interventions for equitable dental care. The purpose of this research is to determine the dental preventive service utilization of CSHCN as compared with CWSHCN using National Survey of Children’s Health 2020 (NSCH, 2020) data.

## Methods

This research was recognized by the West Virginia University Institutional Review Board as non-human subject research (Protocol number 2203535297). The data source for this research is the publicly available NSCH, 2020 [16]. It is routinely used to monitor healthcare services for children. The NSCH consists of a nationally representative sample of non-institutionalized children, ages 0–17 years. It is sponsored by the Maternal and Child Health Bureau of Health Resources and Services Administration (an agency of the U.S. Department of Health and Human Services) Data Resource Center for Child and Adolescent Health. In NSCH, 2020, 42,777 surveys were completed from June 2020 to January 2021 [2]. There was a two-phase collection approach with an initial screener survey followed by a topical survey if there were children present in the home [12]. Questions concerning dental preventive services were available, however, unmet dental need was not queried in NSCH, 2020. Parents/guardians responded to validated questions including one asking them to recall preventive dental services received during the previous year (June 2019 to January 2021). Although sub-setting NSCH data is possible, it is recommended that only statistics with a sample size or unweighted denominator of ≥30 be presented in data reports [13]. Also, when the 95% confidence interval exceeds 20 percentage points or is 1.2 times the estimate (relative standard error is >30%) the data has poor reliability [17].

This study had a cross-sectional study design involving secondary data analysis. The outcome variable of interest was dental preventive service utilization for children ages, ≥1 to <18 years. Participants’ parents were queried over the phone, through the mail, through e-mail, or online during the months of June 2020 to January 2021 as to if their child received a preventive dental “check-up” within the previous 12 months. The response to this yes/no question was operationalized for the variable, dental preventive service utilization. It represents preventive dental service utilization during June 2019 to January 2021.

The key independent variable, CSHCN status (yes/no) was reported in the NSCH, 2020 dataset as an existing variable, SC_CSHCN, with yes/no options. This derived variable provided by NSCH is based upon the CSHCN screener for five questions that meet the Maternal and Child Health Bureaus’ consequences-based definition of CSHCN. Included in the definition is that a child uses/needs prescription medication; has above average use/need for medical/mental/educational services; has functional limitations as compared with peers of the same age; uses/needs specialized services including occupational therapy, physical therapy, or speech therapy; and/or has treatment or counseling for emotional/developmental problems [17]. These consequences must be due to a medical/health condition which had or is expected to have a duration of 12 months [17]. In the public data file, if a child has more than one such need, only one is reported [17]. The definition has had no substantive changes since 2016 [17].

The healthcare utilization theory for this study is the Andersen Behavioral Model of Health Services Use, a widely used model for service utilization research. In the model, contextual and individual predisposing factors (ie., demographic factors, social factors, and beliefs); enabling factors (ie., health policies, financial concerns, and organizational structures); need; and, health behaviors (ie., personal health practices, etc.) result in utilization. Therefore, the research included other available variables in the NSCH, 2020 dataset that epidemiologically fit the Anderson Theoretical Model. These are predisposing factors (sex, age, and race); enabling factors (highest education in the household, insurance coverage); and health behaviors (family member smoking status, and medical care utilization within the previous year). Inclusion criteria were complete data on dental preventive service utilization, CSHCN status, sex, age, race, highest education in household, family member smoking status, and medical care utilization within the previous year (n = 40,140).

SAS (SAS Institute Inc., Cary, NC) version 9.6 was used for the data analyses. The analyses included descriptive statistics of frequencies, Rho-Scott Chi square analyses for group differences, and multivariable logistic regression to compare dental preventive service utilization between CSHCN and CWSHCN. The logistic regression analyses included accounting for the complex survey design by using the topical file’s provided weights (FWC), cluster (HHID) and strata (STRATUM and FIPSST) variables.

## Results

Table 1 provides the details of the description of the sample with weighted percentages. It consisted of 40,140 children, of whom 51.0% were male, and 36.3% were ages 13 years to less than 18 years. There were 67.0% who were non-Hispanic white. Nearly three-fourths (71.3%) of the households had a parent/guardian with education beyond high school graduation. Nearly all the children had insurance access (93.4%). There were 20.6% of the children who were CSHCN. Three-fourths of all children received a dental preventive visit within the past 12 months.

**Table 1:** Sample characteristics, national survey of children’s health, 2020.

Sample characteristic	Frequency	Weighted row percent
**Total Sample**	40140	100%
**Sex**		
Male	20730	51.0%
Female	19410	49.0%
**Age in years**		
Less than 6	10495	28.7%
6 to less than 13	12685	35.1%
13 to less than 18	16960	36.3%
**Race/ethnicity**		
Non-Hispanic white	30842	67.0%
Non-Hispanic black	2915	13.6%
Other	6383	19.3%
**Highest education in household**		
Less than high school	1067	9.5%
High school graduate	5357	19.2%
Some college/technical school	9032	20.6%
College degree/above	24784	50.7%
**Smoking in household**		
Yes	5403	14.4%
No	34737	85.6%
**Insurance coverage**		
Yes	38385	93.4%
No	1755	6.6%
**Medical visit within the past 12 months**		
Yes	33944	81.5%
No	6195	18.5%
**Dental preventive visit within the past 12 months**		
Yes	32167	75.0%
No	7973	25.0%
**Children with Special Healthcare Needs**		
Yes	9723	20.6%
No	30417	79.4%

The insurance coverage category was imbalanced. There were over 90% CSHCN and CWSHCN (95.9% and 92.8% in columnar percentages, respectively) who had insurance coverage. As balancing the data set on insurance through oversampling was not possible, insurance coverage was not included in the further analyses.

Table 2 provides the details of the relationships of the variables of interest with having a dental preventive visit within the past 12 months. Significant relationships with dental preventive visits existed including CSHCN status, the categories of age, race/ethnicity, education in the household, smoking in the household, insurance coverage, and status of medical visit in the past 12 months. There were 78.4% of CSHCN who had a dental preventive visit within the past 12 months, and 74.1% of CWSHCN who had a dental preventive visit within the past 12 months (*p* = 0.0006).

**Table 2:** Bivariate analyses of the outcome, dental preventive visits within the past 12 months, and variables of interest, national survey of children’s health, 2020.

	Dental preventive visit within the past 12 months		No dental preventive visit within the past 12 months		p-value
	Number	Weighted %	Number	Weighted %	
**Sex**					0.2089
Male	16475	74.4%	4255	25.6%	
Female	15692	75.7%	3718	24.3%	
**Age in years**					< .0001
Less than 6	6157	54.0%	4338	46.0%	
6 to less than 13	11246	84.9%	1439	15.1%	
13 to less than 18	14764	82.0%	2196	18.0%	
**Race/ethnicity**					< .0001
Non-Hispanic white	25085	76.9%	5757	23.1%	
Non-Hispanic black	2147	69.2%	768	30.8%	
Other	4935	72.4%	1448	27.6%	
**Highest education in household**					< .0001
Less than high school	692	64.3%	375	35.7%	
High school graduate	3736	68.2%	1521	31.8%	
Some college/technical school	6899	73.3%	2133	26.7%	
College degree/above	20840	80.2%	3944	19.8%	
**Smoking in household**					< .0001
Yes	3947	70.0%	1456	30.0%	
No	28220	75.8%	6517	24.2%	
**Medical visit within the past 12 months**					< .0001
Yes	27821	77.8%	6113	22.2%	
No	4336	62.6%	1860	37.4%	
**Children with Special Healthcare Needs**					0.0006
Yes	8223	78.4%	1500	21.6%	
No	23944	74.1%	6473	25.9%	

The data for preventive dental visit within the past month and CSHCN status were tested for confounding with logistic regression analysis. The unadjusted odds ratio (UOR) associated with CSHCN for having had a preventive dental visit within the past 12 months was 1.29 (95% CI: 1.13, 1.47), *p* = 0.0002, as compared to CWSHCN. The CSHCN adjusted odds ratios (AORs) remained stable controlling in individual analyses of CSHCN status and sex (1.29); CSHCN status and age (1.02); CSHCN status and race/ethnicity (1.30), education (1.29), and smoking (1.29). The adjusted odds ratio (AOR) for CSHCN changed to 0.86 (0.75–0.98) *p* = 0.0258 when controlled for medical visit within the past 12 months.

The data were further examined to determine if the change in the AOR was an effect modification. The CSHCN status categories and Medical visits within the previous 12 months categories were combined to 4 categories of interaction: CSHCN with a medical visit within the previous 12 months; CSHCN without a medical visit within the previous 12 months; CWSHCN and a medical visit within the previous 12 months; and CWSHCN without a medical visit within the previous 12 months.

Table 3 has the results of the logistic regression models on having had a preventive dental visit within the past 12 months with the interaction terms and the other factors. The unadjusted odds ratio (UOR) associated with CSHCN status with a medical visit status was 1.20 (95% CI: 1.04, 1.39), *p* <0.0001, as compared to CWSHCN who also had a medical visit within the past 12 months. However, this reversed to 0.90 (95% CI: 0.76, 1.06), *p* <0.0001 controlling for sex, age, race/ethnicity, highest education in the household, and smoking in the household.

**Table 3:** Logistic regression on having a dental preventive visit within the past 12 months, national survey of children’s health, 2020.

	UOR	95% CI	p-value	AOR	95% CI	p-value
**CSHCN status**						
CSHCN and med visit	1.20	1.04–1.39	< .0001	0.90	0.76–1.06	< .0001
CSHCN and no med visit	0.46	0.34–0.63	0.002	0.29	0.21–0.41	< .0001
CWSHCN and med visit	Ref			Ref		
CWSHCN and no med visit	0.50	0.43–0.57	< .0001	0.38	0.32–0.46	< .0001
**Sex**						
Male				0.91	0.81–1.03	0.1211
Female				Ref		
**Age in years**						
Less than 6				Ref		
6 to less than 13				5.35	4.58–6.23	< .0001
13 to less than 18				6.08	5.21–7.09	< .0001
**Race/ethnicity**						
Non-Hispanic white				Ref		
Non-Hispanic black				0.69	0.59–0.82	0.0011
Other				0.86	0.72–1.03	0.7378
**Highest education in household**						
Less than high school				0.44	0.33–0.59	0.0006
High school graduate				0.54	0.46–0.63	0.0090
Some college/technical school				0.70	0.61–0.80	0.1238
College degree/above				Ref		
**Smoking in household**						
Yes				0.81	0.69–0.80	0.0143
No				Ref		

Abbreviations: UOR = unadjusted odds ratio for the specific factor; AOR = adjusted odds ratio controlled for factors sex, race/ethnicity, smoking, age, and education; CSHCN = children with special healthcare needs; CWSHCN = children without special healthcare needs; med = medical visit within the past 12 months; Ref = reference group.

## Discussion

In this study, parents/guardians of children, ages 1 to ≤ 18 years, were asked about preventive dental visits occurring June 2019-January 2021. Overall, three-fourths of all children in the survey had a preventive dental visit during that period (78.4% for CSHCN and 74.1% for CWSHCN). These results included 9 months prior to the COVID-19 pandemic and 10 months during the early months of COVID-19 before an available vaccine. Researchers have previously reported, with 2016–2018 data, that 84% of CSHCN and 78% of CWSHCN had preventive dental services within the previous year [11], indicating dental visits for both groups decreased and the decrease was more dramatic for CSHCN.

This study had an interaction of CSHCN status and medical visits within the previous year. Having had a medical visit was associated with CSHCN status also having a dental visit while the CSHCN who did not have a medical visit was also less likely to have a dental visit in unadjusted analysis. However, the pattern for CSHCN and the medical visit reversed in adjusted analysis. In adjusted analysis, CWSHCN and a medical visit were the most likely to also have a dental visit during this period.

Previous researchers have noted that unadjusted data indicate associations of two variables with “all accompanying biases/confounds,” whereas an adjusted reversal in an “all-else-equal, statistically based, logic contradicts” the observed UOR [18]. The adjusted results of this study indicate that during the study period CSHCN were less likely to have had a preventive dental visit than CWSHCN with a medical visit within the past 12 months.

In a study of NSCH 2003–2004 data, CSHCN (limited to ages 3 to 5 years) had higher odds of having a preventive dental visit (AOR = 1.26 [95% CI: 1.04, 1.52]) [19]. The differences between the 2003–2004 study and the current study may be explained by the influence of the COVID-19 pandemic on preventive healthcare seeking behavior in addition to the different age groups studied. In research support of the COVID-19 pandemic as an explanation for less healthcare seeking, there are several studies indicating less dental service use in 2020.

Researchers who examined data from March-April 2020, indicated a 12.1% decrease in demand for overall dental visits [15]. The most common dental services that were forgone were preventive and diagnostic services [15]. In another study, caregivers were queried in June and July 2020 about unmet healthcare need that occurred in the previous 3 months [20]. The greatest unmet need was for dental care (16%) [20]. Estimates were that 41.4% of missed visits (approximately 110,000) were not caught up [15].

There are many consequences for missed preventive appointments, especially for children. The most significant consequence is increased use of emergency departments (ED) for dental urgent care needs [15]. Researchers estimated ED use for dental care would increase 0.14–0.2% in the years to come because of foregone routine preventive and diagnostic services [15].

Dental care access is important. Preventive care and minimally invasive interceptive care for incipient lesions makes it possible to manage dental issues before they become major challenges. A goal of dental care is to provide anticipatory guidance to avoid dental caries and the possibility of needing hospitalized care for dental issues. Avoiding general anesthesia is particularly important for CSHCN. However, learning of that need through preventive dental evaluations, and having such treatment available is itself important to avoid even greater systemic consequences.

This research provides a pre-pandemic/early pandemic baseline that will be helpful for future research. It is important to understand the patterns of dental care utilization, particularly for vulnerable populations in times of disruption. The impact of the COVID-19 pandemic was far-reaching. Some parents/guardians may still have qualms about seeking dental care. During the initial months of the COVID-19 pandemic, patients were triaged by telephone; access to dental offices and operatories were restricted as patients were asked to wait in their car to be invited into the facility by phone, or were asked not to enter earlier than 15 minutes before their appointment; and, many ventilation systems were improved. Dentists took measures to decrease aerosols during treatment with the increased use of silver diamine fluoride, pre-treatment rinses, and atraumatic restoration techniques. Safety protocols were reviewed and increased as needed.

It should be noted that the American Dental Association recommended the postponement of elective treatment from March 16, 2020, to May 20,2020; and, as a result, the overall number of dental visits for adults declined by 20% [12]. A similar decline (22%) occurred in Germany where adults postponed a “check-up/regular dental examination [21];” and in Japan when 28.7% of regular dental visits were discontinued during the pandemic [22]. The COVID-19 pandemic affected some nations and communities at higher levels than others (for example, low-income communities in Australia [23]) however, early research did not show a difference in delayed dental care by race/ethnicity or census division in the U.S. [24]. The overall affect upon CSHCN is to be determined.

This study has several strengths. It uses national data that were collected using a representative design. The national study data source has been ongoing since 2003 and uses validated questions. Another strength is that there are sample weights provided to researchers that allow the research to be representative of the nation.

Study limitations include the lack of more granular information to explain the difference in accessing dental care; lack of time-specific data for pre-pandemic and pandemic utilization; lack of data concerning access for unmet dental need; and sample size limitations for finer detailed analyses. Additionally, as the responses were parent/guardian reports, the responses are subject to recall bias and social desirability bias. With the reference period for parents/guardians to consider for their child’s preventive dental visit within the previous 12 months having included the 9 months prior to the COVID-19 pandemic and the 10 months during the early months of COVID-19, it is also possible that the recall was biased by the pandemic.

## Conclusions

CSHCN were less likely to have preventive dental care than CWSHCN when the logistic regression analysis was adjusted for sex, race/ethnicity, age, smoking in the household, medical visits within the year, insurance coverage, and highest level of education in the household. Understanding service use patterns is important for resource allocation to address equity in care.
